# Effect of Fertilization Levels on Growth and Physiological Characteristics of Containerized Seedlings of *Vaccinium oldhamii*

**DOI:** 10.3390/plants14152409

**Published:** 2025-08-04

**Authors:** Da Hyun Lee, Chung Youl Park, Do Hyun Kim, Jun Hyeok Kim, Hyeon Min Kim, Chae Sun Na, Wan Geun Park

**Affiliations:** 1 Warm-Temperate and Subtropical Forest Research Center, National Institute of Forest Science, Seogwipo 63582, Republic of Korea; dahyunlee@korea.kr; 2 Horticultural and Herbal Crop Environment Division, National Institute of Horticultural and Herbal Science, Wanju 55365, Republic of Korea; rtpcr@korea.kr; 3 Forest Bioresources Department, Baekdudaegan National Arboretum, Bonghwa 36209, Republic of Korea; kdh88@koagi.or.kr (D.H.K.); kjh9859@koagi.or.kr (J.H.K.); khm0766@koagi.or.kr (H.M.K.); 4 Division of Forest Science, Kangwon National University, Chuncheon 24341, Republic of Korea

**Keywords:** *Vaccinium oldhamii*, physiological characteristics, growth performance, medicinal plant

## Abstract

*Vaccinium oldhamii*, a blueberry species native to Korea, is a deciduous shrub in the Ericaceae family. Its fruit possesses various pharmacological properties, including anti-inflammatory effects and potential for treating osteoporosis. This study evaluated the effects of five fertilization concentration levels using Multifeed 20 (N:P:K = 20:20:20) on the growth and physiological characteristics of one-year-old *V. oldhamii* container seedlings. Treatments included 0 g·L^−1^ (control), 0.5, 1.0, 1.5, and 2.0 g·L^−1^. Increases in stem thickness, root length, and total dry weight were observed in the control, 0.5, 1.0, and 1.5 g·L^−1^ treatments, whereas growth declined at 2.0 g·L^−1^. Mortality rates exceeded 15% at concentrations above 1.0 g·L^−1^. Photosynthetic capacity and chlorophyll content increased with fertilization. However, while growth improved with increasing fertilizer up to a certain level, it declined at the highest concentration. A fertilization rate of 0.5 g·L^−1^ proved to be the most economically and environmentally efficient for producing healthy seedlings. This study provides the first fertilization threshold for *V. oldhamii*, offering practical guidance for nursery production and forming a foundation for future domestication strategies.

## 1. Introduction

*Vaccinium oldhamii*, an indigenous Korean blueberry, is a deciduous shrub belonging to the genus *Vaccinium* in the Ericaceae family. Plants of the genus *Vaccinium* are valued for their edible fruits and high ornamental value, which has prompted active research and breeding efforts in North America [1].

It typically grows to a height of 1–3 m in mountainous regions. The flowers, ranging from pale pink to greenish-yellow or reddish-brown, bloom between May and June, forming 5–15 pendulous racemes at the tips of new shoots. The fruits, 4–6 mm in diameter, ripen to black color from September to October [2].

These fruits are rich in pharmacologically active compounds, including polyphenols such as anthocyanins, flavonols (e.g., quercetin), and chlorogenic acid, as reported in related *Vaccinium* species. These bioactive compounds show anti-inflammatory efficacy by suppressing NF-κB and MAPK signaling pathways [3], have an antiviral effect by inhibiting influenza virus replication through polyphenols and proanthocyanidins [4], and demonstrate efficacy against leukemia cells by inducing apoptosis through flavonoids [5]. 

The functional food industry relies on diverse plant materials, emphasizing the significance of continuous material supply and quality standardization for successful industrialization. Commercialization of forestry-based biological resources remains challenging due to the lack of reliable material supply and standardized quality indicators [6]. Although native species like *V. oldhamii* are recognized as valuable forestry resources in Korea, their mass propagation remains problematic. To facilitate their industrial application, it is essential to build a comprehensive information system and develop large-scale cultivation technologies.

Nitrogen (N), phosphorus (P), and potassium (K) are essential macronutrients for plant growth. Nutrient deficiencies can increase susceptibility to both biotic and abiotic stressors. Appropriate fertilization improves plant nutrient status, leading to enhanced yield and quality [7]. However, excessive fertilizer use can saturate plants or induce exponential, unsustainable growth, negatively affecting overall performance and secondary metabolite production [8]. Overapplication also reduces nutrient use efficiency and leads to environmental pollution. Thus, effective fertilization practices must balance sufficient nutrient supply with minimal leaching, taking into account species-specific needs, developmental stages, environmental conditions, and soil characteristics [9,10,11].

Two primary propagation techniques are used for *V. oldhamii*: seed sowing in moss during autumn and greenwood cuttings during summer. However, seed germination rates are 10%, and vegetative propagation is seasonally limited, resulting in extremely low propagation efficiency [12]. While prior studies have examined the germination characteristics of *V. oldhamii* seeds [13], research on its cultivation practices is limited.

Container seedlings cultivation requires precise control of environmental factors such as temperature, humidity, light, and carbon dioxide, along with the use of appropriate containers, growth media, irrigation, and fertilization [14,15,16].

Among these, fertilization is a key determinant of seedling growth and quality. Appropriate fertilization can provide the necessary nutrients for a species, resulting in the production of high-quality seedlings [14]. For example, two-year-old *Pinus koraiensis* seedlings achieved maximum biomass at 1.0 g·L^−1^ fertilization [16], and *Sageretia thea* showed optimal growth at 1.0 to 1.5 g·L^−1^ [17].

However, despite its high potential for industrial applications, *V. oldhamii* still faces several practical challenges. First, its low seed germination rate and seasonal limitation in vegetative propagation hinder large-scale production. Second, insufficient basic data on its growth behavior and physiological responses under various environmental conditions have delayed the development of standardized cultivation techniques. Third, difficulties in securing a stable supply of plant material and ensuring consistent quality have limited its industrial standardization. To address these gaps, it is necessary to understand the growth and physiological responses of *V. oldhamii* under controlled conditions to establish optimal cultivation strategies.

This study aimed to determine the optimal fertilization level for container-grown *V. oldhamii* seedlings. Five fertilization concentration levels were tested to evaluate their effects on seedling growth and physiological characteristics. The findings provide fundamental data for efficient seedling production and offer practical insights into optimizing fertilization for large-scale cultivation and industrial application of this native species.

## 2. Materials and Methods

### 2.1. Materials

Fruits of *V. oldhamii* were collected in October 2022 in Buyeo-gun, Chungcheongnam-do, South Korea. The fleshy portion of each fruit was removed using a knife. Prior to experimentation, the seeds were stored in a seed bank at −20 °C with a relative humidity (RH) of 40%. Seeds were soaked in 500 ppm gibberellic acid (GA_3_) for 24 h and sown in Jiffy-7 pots. Sowing was conducted under controlled temperature conditions of 25/15 °C (day/night) in a growth chamber with a 12/12 h light/dark cycle. Sowing was performed on 20 October 2022, and germination commenced on 23 December 2022. Healthy seedlings with true leaves were transferred to a greenhouse for acclimatization. When the roots filled the pots, the seedlings were transplanted into GS150 containers (Diameter 14.5 cm × Height 13.5 cm) for a second transplantation. The growth medium consisted of horticultural soil (Kebi Premium Soil; Kebifarm, Cheonan, Republic of Korea) mixed with perlite (Kebi Perlite; Kebifarm, Cheonan, Republic of Korea) in a 1:0.2 ratio to enhance aeration.

### 2.2. Fertilization Treatment

**Figure 1:**
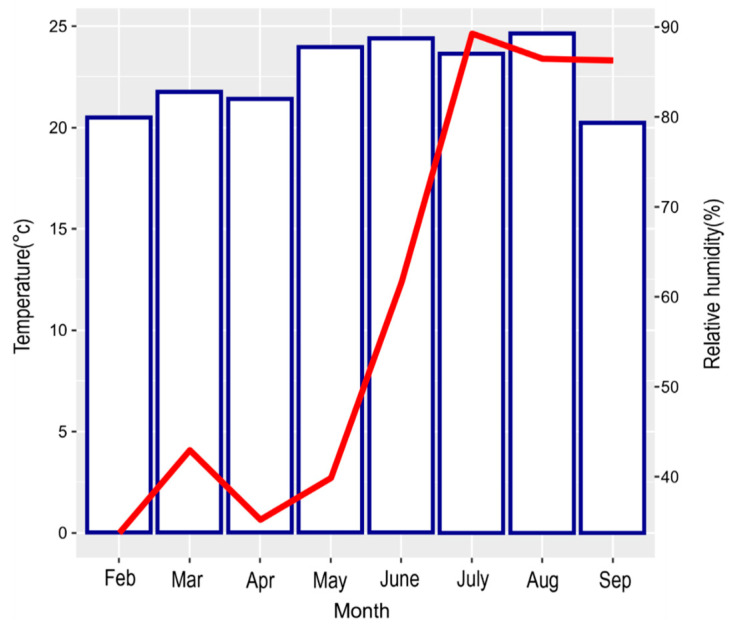
Temperature and relative humidity conditions during the experiment.

Fertilization was applied once a week for a total of 10 weeks, from 3 July 2023 to 4 September 2023, after confirming that the roots had been securely established post-transplantation. Five fertilization concentration levels were applied: untreated control, 0.5 g·L^−1^, 1.0 g·L^−1^, 1.5 g·L^−1^, and 2.0 g·L^−1^. Multifeed 20 (N:P:K = 20:20:20; Haifa Chemical Co., Haifa, Israel), a water-soluble fertilizer containing N, P, K, Mg, Fe, Mn, Zn, Cu, Mo, and B, was used. This fertilizer is widely used in seedling production and commonly applied via fertigation (drenching) and foliar spraying during the vegetative growth stage. The experiment consisted of five fertilization treatments, each with four replicates. In each replicate, 10 seedlings were planted, resulting in 40 seedlings per treatment and a total of 200 seedlings. A volume of 150 mL of fertilizer solution was applied per container at each level. The control group received the same volume of water. Fertilization was conducted between 8 and 10 am or 4 and 6 pm to minimize damage from high temperatures and sunlight. The experiments were conducted in a glass greenhouse at the Forest Environment Research Building of the National Baekdudaegan Arboretum. Air circulation was maintained by operating ventilation fans, and environmental conditions were monitored hourly using a data logger (HOBO, MX1101-01, Onset Computer Corporation, Bourne, MA, USA) to measure temperature and humidity. During the experimental period (February to September 2023), the average temperature and average relative humidity were 22.92 ± 3.27 °C and 57.86 ± 27.40%, respectively (Figure 1). These climatic conditions reflect the typical seasonal pattern of a temperate monsoon climate, with rising temperatures and humidity levels during spring and summer. Temperature and relative humidity data are presented in Figure 1, with the values explicitly indicated for clarity.

### 2.3. Growth Characteristics

The evaluation of container seedling quality followed methods comparable to those used in field seedling assessments. Seedling establishment and growth were predicted by evaluating parameters such as collar diameter, root length, and root collar diameter [15,18]. Seedling specifications for afforestation were based on collar diameter, minimum root collar diameter, and maximum H/D ratio according to seedling form and age. Seedlings meeting the required collar and root collar diameters were deemed acceptable [19]. In this study, collar diameter, root collar diameter, and dry weight were measured to evaluate seedling growth.

To assess the growth response to fertilization, collar and root collar diameters were measured weekly using electronic calipers and steel tape for 40 seedlings per treatment group. The H/D ratio (height [cm]/root collar diameter [mm]) was calculated to assess seedling health [20]. On 12 October 2023, six individuals per treatment group were selected to compare aboveground (leaves and stems) and belowground (roots) growth. These samples were dried in an oven (JSR Co., Ltd., Gong-ju, Republic of Korea) at 70 °C for 72 h. Biomass distribution ratios such as T/R, LWR, SWR, and RWR were calculated from the measured data:

▪ H/D ratio = height (mm)/root collar diameter (mm);
▪ T/R ratio = leaf + shoot dry weight (g)/root dry weight (g);
▪ LWR = leaf dry weight/total dry weight (g);
▪ SWR = stem dry weight/total dry weight (g);
▪ RWR = root dry weight/total dry weight (g).

### 2.4. Photosynthetic Characteristics

To assess the photosynthetic response to fertilization, leaves in the second position from the top of vigorously growing seedlings were selected. A portable photosynthesis system (Li-6800, LI-COR Inc., Lincoln, NE, USA) was used for measurements. The experiment was conducted on 20 September 2023, during active growth. A-Ci curves (response of CO_2_ assimilation to CO_2_ concentration, ranging from 0 to 1400 μmol·m^−2^·s^−1^) were measured. These curves were used to determine various parameters, such as V_cmax_, J_max_, and RD, based on three replicates [21]. During the measurements, chamber conditions were maintained at an airflow rate of 600 μmol·s^−1^ and a temperature of 25 ± 1 °C.

### 2.5. Chlorophyll Content Analysis

To examine chlorophyll content variations among seedlings under different fertilization levels, measurements were recorded on 25 September 2023. Five seedlings per treatment group were selected, and the second and third leaves from the shoot apex were harvested. Chlorophyll was extracted using dimethyl sulfoxide (DMSO) following the method of Hiscox and Israelsta [22]. Leaf samples (50 mg) were placed in vials with 5 mL of DMSO solution and incubated at 65 °C in a constant-temperature chamber (JSGI-10T, JSR Co., Ltd., Republic of Korea) for 6 h. The absorbance of the extracts was measured at 663 and 645 nm using a microplate reader (EPOCH2, BioTek, Shoreline, WA, USA). Chlorophyll a, b, and total chlorophyll contents were calculated, along with the chlorophyll a/b ratio and total chlorophyll (a + b) content [23]:

▪ Chlorophyll a (mg·g^−1^·fresh weight) = (12.7 × A663 − 2.69 × A645);
▪ Chlorophyll b (mg·g^−1^·fresh weight) = (22.9 × A645 − 4.68 × A663);
▪ Total chlorophyll (mg·g^−1^·fresh weight) = (8.02 × A663 + 20.20 × A645).

### 2.6. Statistical Analysis

Results are expressed as mean values ± standard error. R software (version 4.3.1) (http://www.r-project.org, accessed on 25 October 2023)was used for statistical and correlation analyses. One-way analysis of variance (ANOVA) was used for data analysis, and differences between means were evaluated at the 5% significance level through post hoc analysis using Duncan’s multiple range test (*p* < 0.05).

## 3. Results and Discussion

### 3.1. Growth Characteristics

#### 3.1.1. Shoot and Root Growth

Shoot and root growth of *V. oldhamii* seedlings differed substantially among the fertilization treatments (Table 1). Shoot growth was generally greater in the control, 0.5 g·L^−1^, and 1.5 g·L^−1^ treatment groups, while the 2.0 g·L^−1^ group exhibited the lowest shoot growth. The control group showed the highest relative shoot growth, followed by the 0.5 g·L^−1^, 1.0 g·L^−1^, 1.5 g·L^−1^, and 2.0 g·L^−1^ treatment groups, respectively.

Root growth was comparable among the control, 0.5 g·L^−1^, 1.0 g·L^−1^, and 1.5 g·L^−1^ treatment groups, whereas the 2.0 g·L^−1^ group recorded the lowest root growth. Relative root growth was significantly enhanced in the 0.5 g·L^−1^ and 1.0 g·L^−1^ groups, followed by the 1.5 g·L^−1^, 2.0 g·L^−1^, and control groups.

**Figure 2:**
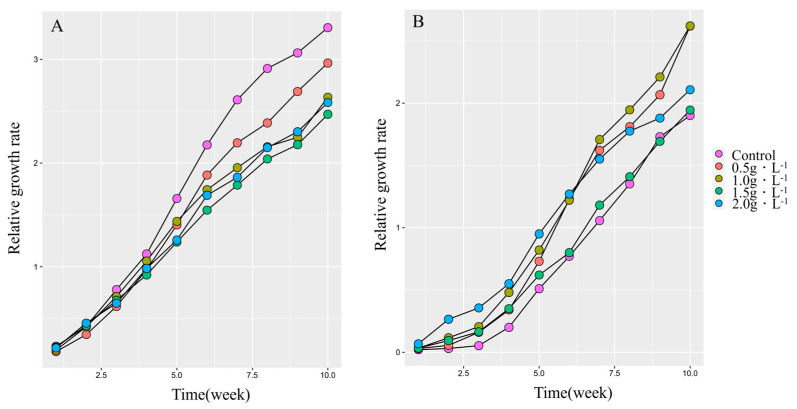
Trends in height growth (**A**) and root collar diameter (**B**) in *V. oldhamii* treated with fertilizer over 11 weeks.

During the 15-week growth period, *V. oldhamii* container seedlings exhibited a steady increase in both shoot and root growth rates beginning in week 5 (Figure 2). Growth was most pronounced in the control and 0.5 g·L^−1^ treatment groups, while the 2.0 g·L^−1^ group displayed impaired growth. These results suggest that nutrient supplementation positively affected seedling development, with lower fertilizer concentrations promoting better growth. Conversely, the reduced growth in the 2.0 g·L^−1^ treatment indicates that excessive fertilization can be detrimental. Similar findings were reported by Eo et al. [17] in *S. thea* container seedling, where excessive fertilization at 2.0 g·L^−1^ led to growth inhibition. These observations underscore the importance of optimizing fertilization levels to enhance growth while avoiding adverse effects.

#### 3.1.2. Height-to-Diameter (H/D) Ratio

The height-to-diameter (H/D) ratio is a key indicator of seedling taper. Higher H/D ratios can suggest increased susceptibility to environmental stressors, such as wind and drought, requiring careful consideration during planting [19]. The highest H/D ratio was observed in the 2.0 g·L^−1^ treatment group, while the 0.5 g·L^−1^ and 1.0 g·L^−1^ groups showed relatively lower values (Table 1). All *V. oldhamii* seedlings across treatment groups fell within the typical H/D range for conifer container seedlings (1–0), reported as 13.5 ± 4.3 (5–22) cm·mm^−1^ [20]. This indicates that the balance between aboveground and belowground growth was generally maintained regardless of fertilization level. Similar results were reported by Choi et al. [24] for *Pinus densiflora*, where all treatment groups, including the control, exhibited comparable H/D ratios ranging from 5.5 to 5.8 cm·mm^−1^. Mortality was tracked during the fertilization period, with the highest mortality occurring in the 2.0 g·L^−1^ treatment group. Lower mortality rates were recorded in the control and 0.5 g·L^−1^ groups. Seedling death was typically preceded by marginal leaf browning followed by wilting, indicating that the highest fertilizer level caused the most severe physiological stress. Excessive fertilization can increase soil salinity, disrupt beneficial soil microbial activity, and, without adequate irrigation, induce water stress [19,25,26]. These findings highlight the need for proper fertilization management, as excessive nutrient input can negatively affect seedling health and survival.

### 3.2. Biomass and Top/Root Ratio (T/R)

Biomass accumulation is strongly correlated with root collar diameter and is a key factor influencing seedling survival and growth at afforestation sites [27]. The ratio between aboveground and belowground biomass—referred to as the top/root (T/R) ratio—is a crucial measure for evaluating seedling balance and transplant potential [28].

In this study, biomass measurements were taken at the end of the 15-week post-fertilization period. Significant differences in biomass among treatments were observed (Table 2). The 1.5 g·L^−1^ treatment group recorded the highest total biomass (0.81 ± 0.39 g), while the control, 0.5 g·L^−1^, and 1.0 g·L^−1^ groups showed no significant differences. The 2.0 g·L^−1^ group exhibited the lowest total and root biomass, though only root biomass differed significantly.

The T/R ratio was highest in the 2.0 g·L^−1^ group (4.59 ± 1.58 g·g^−1^) and lowest in the control group (1.67 ± 0.14 g·g^−1^). The T/R ratio tended to increase with rising fertilizer levels, although no significant differences were noted between the 0.5 g·L^−1^ and 1.0 g·L^−1^ treatments. This pattern suggests that higher fertilizer concentrations and suppressed root development relative to shoot growth result in unbalanced biomass allocation. As noted by Aranda et al. [29] and Cho et al. [30], seedlings with high T/R ratios and excessive aboveground biomass are more prone to water stress, which can reduce establishment success during afforestation.

#### Biomass Allocation

**Figure 3:**
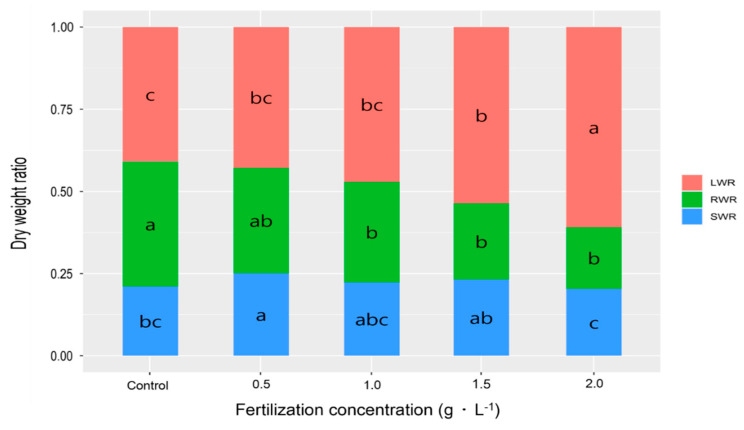
Effect of various fertilizer concentrations on the dry weight ratio of *V. oldhamii* container seedlings. Different letters indicate values significantly different according to Duncan’s multiple range test at the 5% level (*p* < 0.05).

To assess the effects of various fertilization levels on biomass allocation in one-year-old *V. oldhamii* seedlings grown in containers, the leaf weight ratio (LWR), stem weight ratio (SWR), and root weight ratio (RWR) were calculated using the biomass values for each plant part (Figure 3).

The LWR increased with fertilization level, peaking at 0.61 ± 0.07 g·g^−1^ in the 2.0 g·L^−1^ treatment group, while the control group had the lowest LWR at 0.41 ± 0.02 g·g^−1^. The SWR was highest in the 0.5 g·L^−1^ treatment group (0.26 ± 0.01 g·g^−1^) and lowest in the 2.0 g·L^−1^ group (0.21 ± 0.04 g·g^−1^), with no consistent trend observed across treatments. The RWR was highest in the control group (0.39 ± 0.03 g·g^−1^) and lowest in the 2.0 g·L^−1^ group (0.19 ± 0.05 g·g^−1^).

These results indicate that higher fertilization levels in *V. oldhamii* container seedlings led to increased leaf biomass and reduced root biomass. This trend reflects the plant’s adaptive response to nutrient-rich conditions, consistent with general patterns of physiological and morphological adaptability in response to environmental factors [31].

### 3.3. Photosynthetic Characteristics

To evaluate the photosynthetic response of *V. oldhamii* to different fertilization levels, A-Ci curves were analyzed to determine the maximum carboxylation rate (V_cmax_), maximum electron transport rate (J_max_), and dark respiration (RD). Photosynthetic capacity is governed by the balance between RuBisCO (ribulose-1,5-bisphosphate carboxylase/oxygenase) activity and ribulose-1,5-bisphosphate (RuBP) regeneration, the latter of which is limited by electron transport efficiency [32,33].

**Figure 4:**
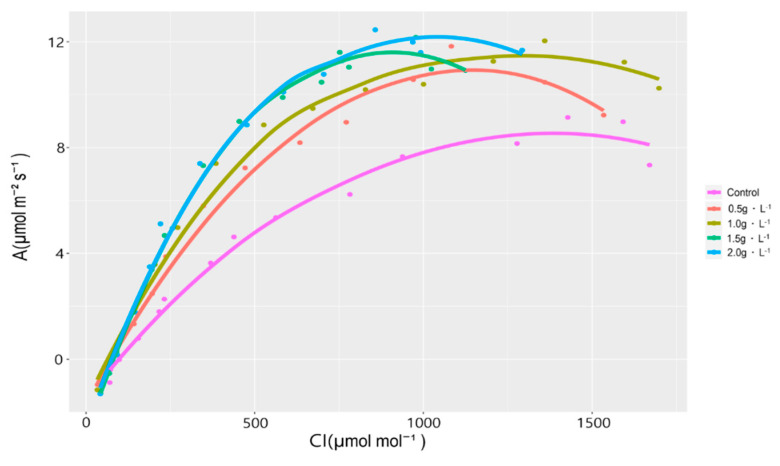
A-Ci curves of *V. oldhamii* grown under five different fertilization treatments.

**Figure 5:**
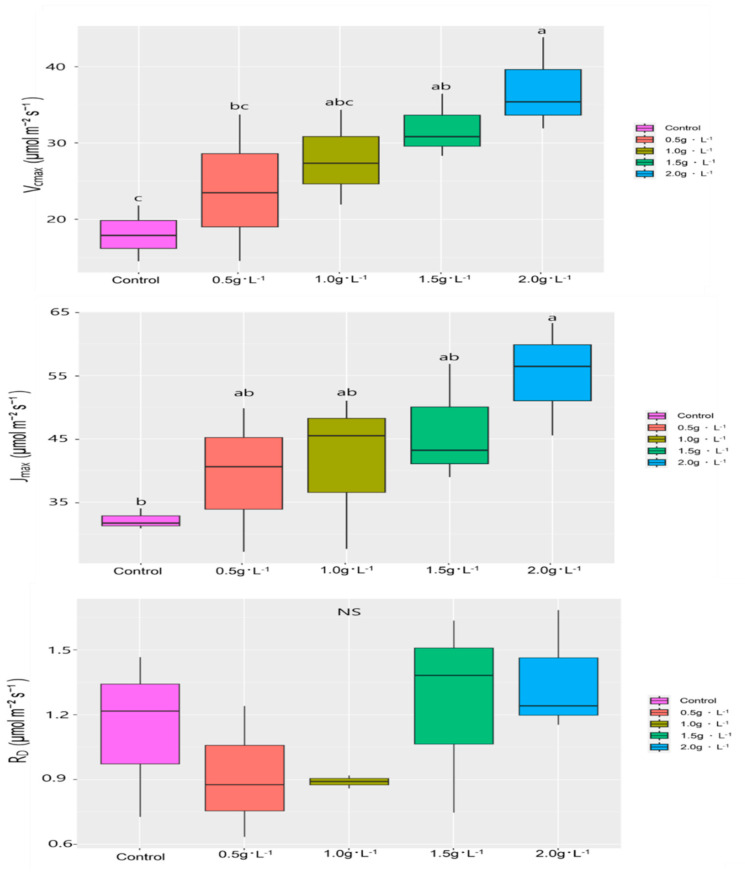
Photosynthetic parameters of *V. oldhamii* grown under five different fertilization treatments. Different lowercase letters (a, b, c) indicate a statistically significant difference (Duncan’s multiple range test, *p* < 0.05), while NS indicates no statistical difference.

The A-Ci curve analysis revealed that both V_cmax_ and J_max_ increased with higher fertilization levels (Figure 4 and Figure 5). The control group exhibited a V_cmax_ of 18.05 ± 3.66 mmol CO_2_ m^−2^s^−1^, while the 2.0 g·L^−1^ group reached 37.05 ± 6.15 mmol CO_2_ m^−2^s^−1^, nearly doubling the photosynthetic capacity. A significant increase in V_cmax_ was observed as the fertilization level increased. J_max_ was also highest in the 2.0 g·L^−1^ treatment group. Although no significant differences were detected among the 0.5, 1.0, and 1.5 g·L^−1^ treatments, mean values showed an increasing trend. No significant variation in RD was observed among treatments.

These findings suggest that fertilization enhances the photosynthetic capacity of *V. oldhamii*. However, increased photosynthetic efficiency did not translate directly into greater growth; the 2.0 g·L^−1^ group exhibited reduced development. Similar trends were reported in studies on *S. thea* and *Machilus thunbergii* by Eo et al. [17] and Sung [34], where photosynthetic traits peaked before declining at excessive fertilization levels. The differing results in *V. oldhamii* highlight the species-specific nature of physiological responses.

Consequently, tailored fertilization strategies are essential to align with the physiological and growth characteristics of container-grown *V. oldhamii*. The divergence between photosynthetic capacity and biomass accumulation underscores the complexity of seedling growth dynamics [35].

### 3.4. Chlorophyll Content

Chlorophyll is critical in plant photosynthesis, enabling light absorption in chloroplasts and conversion into organic compounds. Chlorophyll a and b absorb light at different wavelengths [36].

To investigate the effects of fertilization on chlorophyll content in containerized *V. oldhamii* seedlings, the uppermost two to three leaves were analyzed. Chlorophyll content increased with fertilization level (Table 3). Seedlings exposed to higher fertilization rates exhibited increased levels of chlorophyll a and b, total chlorophyll (a + b), and a higher chlorophyll ratio (a/b). The highest chlorophyll a contents were observed in the 1.5 g·L^−1^ and 2.0 g·L^−1^ groups, at 15.20 ± 0.91 and 14.54 ± 0.76 mg/g^−1^, respectively. Chlorophyll b content was highest in the 1.5 g·L^−1^ group at 4.66 ± 0.12 mg/g^−1^. Total chlorophyll (a + b) content peaked in the 1.5 and 2.0 g·L^−1^ treatments, at 19.86 ± 0.49 and 18.90 ± 1.14 mg/g^−1^, respectively. The chlorophyll a/b ratio was also higher in these groups, with no significant differences observed at lower fertilization levels.

These findings are consistent with earlier studies on *S. thea* and *Hovenia dulcis* by Eo et al. [17] and Lee [37], which showed that higher fertilization increased chlorophyll content, as measured by SPAD values. The trend observed in *V. oldhamii* confirms that increased nutrient availability enhances chlorophyll synthesis.

This study investigated the effects of five fertilization levels (control, 0.5 g·L^−1^, 1.0 g·L^−1^, 1.5 g·L^−1^, 2.0 g·L^−1^) on the growth and physiological characteristics of one-year-old container-grown *V. oldhamii* seedlings. The aim was to identify an optimal fertilization strategy for the large-scale production of healthy, high-quality seedlings.

The study findings indicated that fertilization levels positively influenced growth parameters such as height, root collar diameter, and total dry weight. These parameters showed an increasing trend across the control, 0.5 g·L^−1^, 1.0 g·L^−1^, and 1.5 g·L^−1^ treatment groups. However, the 2.0 g·L^−1^ treatment group exhibited reduced growth. Additionally, as fertilization levels increased, a shift in nutrient allocation was observed, favoring aboveground over belowground components, as evidenced by increased leaf mass and decreased root mass. The control group recorded the highest relative height growth, while the 0.5 g·L^−1^ and 1.0 g·L^−1^ treatments resulted in the highest relative root collar diameter growth. The 1.5 g·L^−1^ treatment group exhibited the highest total dry weight. However, fertilization levels of 1.0 g·L^−1^ and above led to leaf wilting and dieback, indicating that excessive fertilization may cause physiological disorders.

Moreover, indicators of photosynthetic efficiency, such as V_cmax_ and J_max,_ increased with higher fertilization levels. Chlorophyll content also rose with increasing fertilization. Nevertheless, despite the higher photosynthetic efficiency and chlorophyll content observed in the 2.0 g·L^−1^ treatment group, plant growth was comparatively lower, indicating that excessive fertilization may negatively affect development. This discrepancy between enhanced photosynthetic capacity and actual plant growth indicates that increased photosynthesis does not necessarily lead to higher biomass production.

Therefore, for the mass cultivation of *V. oldhamii*, it is crucial to determine precise fertilizer application rates that meet the plant’s nutritional needs while minimizing the risk of soil contamination. These findings suggest that applying low concentrations (e.g., 0.5 g·L^−1^) can reduce *V. oldhamii* seedling mortality, indicating an optimal fertilization range that balances environmental and economic considerations.

This study confirms the potential of *V. oldhamii* as a cultivated species and proposes the standardization of seedling supply and management techniques. Such standardization supports the development of an efficient production system and the use of *V. oldhamii* as a functional material resource.

## 4. Conclusions

This study revealed that varying fertilization levels significantly affect the growth and physiological responses of container-grown *V. oldhamii* seedlings. Among the treatments, seedlings fertilized with 0.5 g·L^−1^ demonstrated superior performance in parameters such as root collar diameter growth and overall survival compared to treatments with higher concentrations. While photosynthetic efficiency indicators such as Vcmax and Jmax, as well as chlorophyll content, increased with higher fertilization, excessive application (≥1.0 g·L^−1^) resulted in leaf wilting, dieback, and reduced biomass accumulation. This indicates a disconnect between physiological performance and biomass accumulation under excess fertilization.

Furthermore, increased fertilization led to a shift in biomass allocation, favoring aboveground growth at the expense of root development, which may compromise seedling stability and field performance.

Therefore, applying a low fertilizer concentration of 0.5 g·L^−1^ may serve as an optimal and economically efficient strategy for enhancing the growth, physiological balance, and survivability of *V. oldhamii* seedlings, while minimizing environmental impact. Although 1.0 g·L^−1^ showed relatively high physiological responses, the 0.5 g·L^−1^ treatment offered better trade-offs in terms of cost, seedling viability, and balanced growth.

A limitation of this study is that finer fertilization increments, such as 0.75 g·L^−1^, were not tested. Future studies should explore these intermediate levels to more precisely determine the optimal fertilization threshold for *V. oldhamii* seedling production.

These findings provide a foundation for standardizing seedling production practices for *V. oldhamii* as a valuable functional plant resource.

## Figures and Tables

**Table 1:** Root collar diameter, height, and height (H)/diameter (D) ratio of *Vaccinium oldhamii* under various fertilization treatments.

Fertilization Concentration (g·L^−1^)	Height		Root Collar Diameter		H/D (cm·mm)
	Growth (cm)	Relative Growth Rate (%)	Growth (mm)	Relative Growth Rate (%)	
Control	12.36 ± 0.78 a	3.30 ± 0.19 a	1.78 ± 0.08 a	1.90 ± 0.14 b	7.05 ± 0.44 bc
0.5	11.58 ± 0.75 a	2.96 ± 0.19 ab	1.89 ± 0.1 a	2.62 ± 0.20 a	6.17 ± 0.21 c
1.0	10.56 ± 0.74 ab	2.64 ± 0.18 b	1.74 ± 0.1 a	2.62 ± 0.24 a	6.45 ± 0.39 c
1.5	11.7 ± 0.79 a	2.47 ± 0.19 b	1.72 ± 0.13 a	1.94 ± 0.24 ab	7.93 ± 0.65 ab
2.0	9.04 ± 0.59 b	2.58 ± 0.19 b	1.24 ± 0.11 b	2.11 ± 0.28 ab	8.53 ± 0.57 a

Each value represents the mean ± SE (standard error). Different letters indicate values that are significantly different according to Duncan’s multiple range test at the 5% level (*p* < 0.05).

**Table 2:** Effect of fertilization levels on dry mass, top/root (T/R) ratio, leaf weight ratio (LWR), stem weight ratio (SWR), and root weight ratio (RWR) in *V. oldhamii*.

Fertilization Concentration (g·L^−1^)	Biomass (g, Dry Weight)				T/R Ratio (g·g^−1^)
	Leaves	Shoot	Root	Total	
Control	0.34 ± 0.04 b	0.18 ± 0.02 b	0.31 ± 0.02 a	0.68 ± 0.04 b	1.67 ± 0.14 c
0.5	0.36 ± 0.04 b	0.21 ± 0.02 b	0.28 ± 0.05 a	0.69 ± 0.11 b	2.28 ± 0.35 bc
1.0	0.40 ± 0.09 b	0.19 ± 0.07 b	0.27 ± 0.10 a	0.65 ± 0.23 b	2.36 ± 0.72 bc
1.5	0.67 ± 0.25 a	0.34 ± 0.16 a	0.35 ± 0.13 a	0.81 ± 0.39 a	3.68 ± 0.41 b
2.0	0.31 ± 0.22 b	0.12 ± 0.10 b	0.11 ± 0.12 b	0.31 ± 0.34 b	4.59 ± 1.58 a

Values represent mean ± SE. Different letters indicate values that are significantly different according to Duncan’s multiple range test at the 5% level (*p* < 0.05).

**Table 3:** Chlorophyll content in *V. oldhamii* grown under five different fertilization treatments.

Fertilization Concentration (g·L^−1^)	Chl (mg/g^−1^)			Chlorophyll a/b
	A	B	a + b	
Control	11.32 ± 0.71 b	3.94 ± 0.22 b	15.25 ± 0.93 b	2.87 ± 0.03 b
0.5	9.13 ± 0.49 c	3.20 ± 0.14 c	12.32 ± 0.63 c	2.84 ± 0.04 b
1.0	8.74 ± 0.58 c	3.26 ± 0.15 c	11.99 ± 0.61 c	2.73 ± 0.17 b
1.5	15.20 ± 0.91 a	4.66 ± 0.12 a	19.86 ± 0.49 a	3.26 ± 0.01 a
2.0	14.54 ± 0.76 a	4.36 ± 0.23 ab	18.90 ± 1.14 a	3.31 ± 0.05 a

Values represent mean ± SE. Different letters indicate values that are significantly different according to Duncan’s multiple range test at the 5% significance level (*p* < 0.05).

## Data Availability

Data are contained within the article.

## Abbreviations

The following abbreviations are used in this manuscript:

H/D ratio	Height-to-diameter ratio
T/R ratio	Top/Root ratio
LWR	Leaf weight ratio
SWR	Shoot weight ratio
RWR	Root weight ratio
DMSO	Dimethyl sulfoxide
V_cmax_	Maximum carboxylation rate
J_max_	Maximum electron transport rate
RD	Dark respiration
RuBP	Ribulose-1,5-bisphosphate
